# Precise Recognition of Adulterated Sliced Mutton Using Machine Vision on Mobile Phone Images

**DOI:** 10.3390/foods15142473

**Published:** 2026-07-13

**Authors:** Yue Huang, Yinghao Gao, Xudong Luo, Menglong Guo, Shuting Cui, Yue Li, Yuchen Zhu

**Affiliations:** 1 College of Food Science, Xizang Agricultural and Animal Husbandry University, Nyingchi 860000, China; 2 College of Food Science and Nutritional Engineering, China Agricultural University, Beijing 100083, China

**Keywords:** sliced mutton, image classification, machine vision, deep learning, mobile phone images

## Abstract

In recent years, the authenticity of sliced mutton has become a growing concern due to the incorporation of non-mutton ingredients and the increasing use of processed and reconstituted meat products. In this study, a low-cost and non-destructive authentication method integrating smartphone-based image acquisition with machine learning and deep learning techniques was developed for the identification of real, processed, and reconstituted sliced mutton. A total of 600 images were collected under standardized conditions, from which color features in RGB, HSV, and Lab color spaces and texture features derived from the gray-level co-occurrence matrix (GLCM) were extracted. Statistical analyses, including the Kruskal–Wallis test, Dunn’s post hoc test, and principal component analysis, demonstrated significant inter-class differences and confirmed the discriminative capability of the extracted features. Four machine learning models (KNN, LDA, RF, and SVM) and three transfer learning-based convolutional neural networks (VGG16, ResNet50, and InceptionV3) were subsequently developed and evaluated. Among the machine learning models, SVM achieved the best classification performance, while VGG16 demonstrated the highest deep learning performance with an accuracy of 96.42 ± 0.51%. Misclassification analysis indicated that processed sliced mutton represented the primary source of classification ambiguity because of its overlapping visual characteristics with both real and reconstituted products. Furthermore, Grad-CAM visualization revealed that the CNN model focused predominantly on texture and structural regions closely associated with muscle and fat distribution, providing interpretability for the classification results. These findings suggest that smartphone-acquired RGB images combined with machine learning and deep learning methods may serve as a promising, low-cost screening approach for preliminary sliced mutton authentication in market surveillance and supply chain inspection.

## 1. Introduction

Mutton plays a significant role in human nutrition due to its high protein content and favorable fat composition. Driven by increasing consumer awareness of healthy diets, the demand for mutton has continued to rise steadily in recent years [1]. However, the high price of mutton leads to high production costs for pure products, such as real sliced mutton. Some businesses use mutton scraps combined with food additives to manufacture processed sliced mutton. Others replace mutton with low-cost meats, such as duck, and incorporate sheep tail fat to produce reconstituted sliced mutton. While these products pose no direct health risk, their production costs are significantly lower than those of real sliced mutton. Selling these visually similar products as real sliced mutton violates consumer rights and disrupts market order. Therefore, it is of great significance to develop rapid and practical screening methods to identify real, processed, and reconstituted sliced mutton.

Recently, mutton quality detection primarily targets biomolecular features, including nucleic acids, fats, and proteins. Standard techniques encompass DNA-based polymerase chain reaction (PCR), protein-based electrophoresis, immunoassay, and metabolite-based chromatography [2,3]. Despite their high accuracy, these methods involve expensive instrumentation and labor-intensive sample preparation. Such limitations hinder their application in rapid, large-scale supply chain screening [4]. Recent advancements in computer vision and deep learning have popularized machine vision-based food inspection. This approach shows significant potential in meat adulteration detection, variety identification, and quality assessment [5,6].

Machine vision captures image or spectral information through various modalities, including visible light, infrared, hyperspectral, and Raman imaging. Among these, visible light imaging systems are widely used for meat quality inspection due to their low cost, portability, and non-destructive nature [7,8,9]. These systems operate in the 400–700 nm range to acquire surface color and texture data. By capturing light reflectance in red (R), green (G), and blue (B) bands, they generate color images for appearance analysis. Early RGB systems typically integrated traditional machine learning for tasks such as meat classification, adulteration detection, and tenderness grading. RGB color histograms and gray-level co-occurrence matrices (GLCMs) are common tools for color and texture feature extraction. These descriptors are often coupled with classifiers, including support vector machines (SVMs), linear discriminant analysis (LDA), and shallow neural networks. In meat classification tasks, Asmara et al. [10] utilized color and texture features with a back-propagation neural network (BPNN) to differentiate pork from beef. Their results highlighted the critical role of GLCM-derived texture features, achieving 89.57% accuracy. This approach is particularly suitable for rapid, low-cost binary classification tasks. Compared to distinguishing between two meat species, identifying adulteration in meat products has greater practical significance. This task also places higher demands on model performance. Song et al. [8] developed a low-cost smartphone video method to detect pork adulteration in beef. By analyzing video data under varying illumination conditions, they used partial least squares regression to predict adulteration levels, reporting *R*^2^ values of 0.73–0.98 and RMSE values of 0.04–0.16 for the predicted adulteration proportion. Similarly, Rady et al. [7] achieved 99.1% accuracy in distinguishing pure beef from adulterated samples using RGB imaging with LDA and ensemble models. However, identifying specific adulterants, such as soy protein, wheat protein, or chicken, resulted in a significant accuracy drop to 48.9–76.1%. Even after feature selection, the accuracy reached only 63.8%. This study further noted that GLCM texture features provided superior discriminative power compared to RGB color features for classification and regression models. Regarding quantitative analysis, Rady reported a maximum correlation coefficient of 98.0% for predicting pork levels in beef using regression trees. Yet, prediction accuracy decreased to 87.0% when incorporating two or more adulterants. Therefore, quantitative adulteration analysis relying solely on RGB color and texture features remains challenging.

Meat quality grading based on color, freshness, and tenderness is achievable through RGB imaging and machine learning [11]. In pork color classification, color and texture features are commonly extracted and processed using methods such as PCA and SVM. Studies have demonstrated that SVM models based on image color features can achieve satisfactory classification accuracy, with overall prediction performance exceeding 70% in pork color grading tasks. In freshness assessment, Jiang et al. [12] employed six color features from RGB and HSV color spaces with a Multi-Layer Perceptron (MLP) neural network. This model classified fresh, semi-fresh, and spoiled beef with approximately 90% accuracy. Subsequently, Li et al. [13] proposed an optimized backpropagation neural network (BPNN) model, in which a metaheuristic algorithm was employed to improve parameter optimization. By integrating color feature information and multivariate analysis, the method achieved high classification accuracy in chilled beef freshness evaluation, significantly enhancing overall prediction performance. In tenderness prediction, using the marbling characteristics from pork loin images in a support vector machine (SVM) yielded a prediction accuracy of only 75%; in contrast, using color features as inputs increased the accuracy to 92.5% [14]. For beef tenderness assessment, connective tissue-related structural features were extracted using machine vision and analyzed using statistical methods such as Principal Component Analysis (PCA) and regression models [15]. These approaches effectively reduced feature dimensionality and established relationships between structural parameters and tenderness, achieving high classification accuracy and significantly improving prediction performance.

Traditional machine learning methods show limited performance with low-level adulteration or high morphological variance. Consequently, integrating RGB imaging with deep learning models, such as Convolutional Neural Networks (CNNs), has become a preferred approach to improve detection accuracy [16]. Bai et al. [17] used smartphone-captured mutton images and a modified CBA M-Invert-ResNet model to achieve high-precision detection of pork proportions (R^2^ = 0.9589). This method significantly outperformed standard CNNs in feature extraction efficiency and is particularly effective for complex feature extraction. Similarly, Setiadi et al. combined RGB imaging with Deep Neural Networks (DNNs) to detect low-value meats, such as pork and poultry, in beef. Compared to the method by Bai et al., Setiadi et al. [18] adopted a neural network architecture with fewer parameters, reducing the computational load by 30%. This lightweight model exhibited superior performance, achieving a classification accuracy of 99.33% and a precision of 98.68%, thereby demonstrating enhanced practical efficiency. RGB imaging combined with deep learning also achieves high recognition accuracy for non-meat adulterants. Smartphone-captured RGB images of beef were analyzed using the Swin Transformer for image classification. By incorporating transfer learning to optimize model performance, this approach successfully detected hydrocolloid adulteration in beef [9]. The results showed a classification accuracy of 99.7% for distinguishing different hydrocolloid types with a detection speed of 3.2 ms. Compared to electrochemical impedance spectroscopy, accuracy improved by 20.5% and testing time was reduced by 99.99%. This method significantly outperforms traditional spectroscopic analysis. Furthermore, the compact model size is suitable for deployment on smartphone devices.

Compared to meat grading or breed classification, detecting adulteration in commercial meat products requires identifying more hidden and subtle feature differences. This challenge places higher demands on feature extraction and model training. In traditional machine learning, feature extraction primarily utilizes methods such as Gray-Level Co-occurrence Matrix (GLCM), color histograms, and Principal Component Analysis (PCA) to extract hand-crafted color, texture, and morphological features [19]. This study therefore developed a low-cost and non-destructive machine vision method based on smartphone-acquired images for distinguishing real, processed, and reconstituted sliced mutton. Handcrafted color and texture features were extracted for conventional machine learning analysis, whereas transfer learning-based convolutional neural networks were constructed using ROI images. In addition to classification performance evaluation, statistical feature analysis, principal component analysis, misclassification interpretation, and Grad-CAM visualization were employed to improve the reliability and interpretability of the proposed screening framework.

## 2. Materials and Methods

### 2.1. Materials and Samples

Sliced mutton samples from nine different brands were purchased from online platforms. These samples were categorized into three types based on the executive standards and ingredient lists indicated on the packaging. Real sliced mutton contained only mutton and followed the GB 2707-2016 standard (National Food Safety Standard: Fresh and Frozen Livestock and Poultry Products). Processed sliced mutton was manufactured from mutton, water, and food additives, adhering to the SB/T 10379-2012 (Quick-frozen Preconditioned Foods) or SB/T 10482-2008 (Quality and Safety Requirements for Prepared Meat Foods) standards. Reconstituted sliced mutton was formulated using duck meat, sheep fat, water, and food additives, following the same SB/T standards. The classification criteria are summarized in Table 1.

Sample categories were assigned based on a combination of product labeling information, ingredient declarations, processing descriptions, and corresponding execution standards provided by the manufacturers. Products containing only mutton as the declared ingredient were categorized as real sliced mutton. Products containing additional ingredients or processing-related additives were categorized as processed sliced mutton, whereas products explicitly labeled as reconstituted or restructured meat products were categorized as reconstituted sliced mutton. No independent molecular or chemical verification was performed; therefore, category assignments reflect manufacturer-declared commercial classifications.

### 2.2. Machine Vision System

A custom machine vision system was developed, comprising a camera, light sources, a background plate, and a computer. Images were captured using an iPhone 15 rear dual-camera system with an IMX803 sensor (aperture f/1.6, focal length 6 mm, Sony Semiconductor Solutions Corporation, Atsugi-shi, Japan). To ensure stable lighting and avoid color contamination, two cool white LED bulbs were fixed within a rectangular photography box. Background color significantly affects image contrast and subsequent Region of Interest (ROI) extraction. Black backgrounds absorbed most light passing through the thin mutton slices, reducing color contrast and resulting in dim, blurred images. In contrast, white backgrounds reflected light, making the red color distinct even in thin slices. However, the fat areas were difficult to extract due to their similarity to the background color. Therefore, a white PP board (150 mm × 200 mm × 3 mm) placed over black cardboard was selected as the background to highlight the mutton slice and improve image quality.

**Figure 1:**
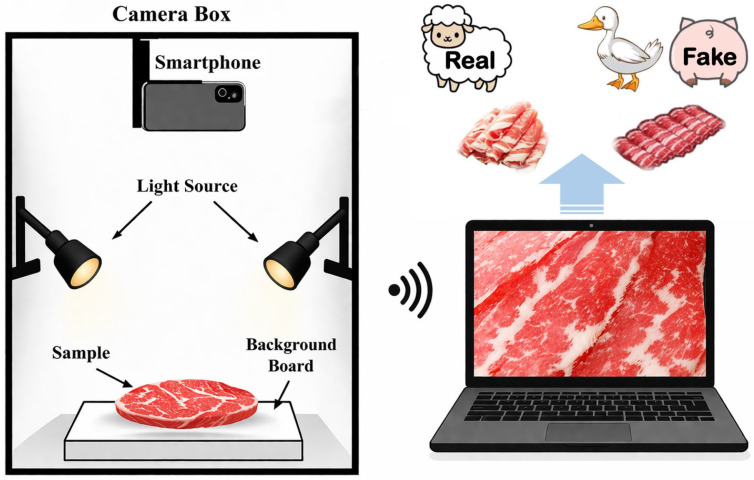
Photograph and schematic illustration of the smartphone-based image acquisition system used in this study.

The machine vision system layout is illustrated in Figure 1. The camera was positioned at the top of the photography box, with light sources on both sides and the PP board directly below. The lens was parallel to the PP board at a fixed vertical distance of 18 cm. This configuration ensured the entire background plate remained within the frame, preventing any partial loss of the mutton slice images.

### 2.3. Image Preprocessing

Prior to image acquisition, frozen mutton rolls were thawed on white polypropylene (PP) boards and carefully unfolded using cotton swabs. The integrity of muscle and adipose tissues was preserved throughout sample preparation. Flattened sliced mutton samples were then placed in the imaging chamber and photographed individually under standardized illumination conditions. Images were acquired using a smartphone camera and saved in JPG format with a resolution of 5712 × 4284 pixels. A total of 600 images were collected, including 200 images each of real, processed, and reconstituted sliced mutton.

The original images contained substantial background information and exhibited considerable variation in image size. Therefore, image preprocessing was performed before feature extraction and model development. For the conventional machine learning workflow, regions of interest (ROIs) containing representative sliced mutton tissues were manually delineated and extracted using the OpenCV library in Python (Version 4.13.0). Irrelevant background regions were removed during ROI extraction. The extracted circular ROIs were subsequently centered on a 3000 × 3000 pixel black background to facilitate image standardization. Handcrafted color and texture features were then extracted from the ROI images for statistical analysis and machine learning model development. ROI extraction was performed manually by a single trained operator according to a predefined circular selection protocol. All images were processed using identical extraction criteria to minimize operator-dependent variability.

**Figure 2:**
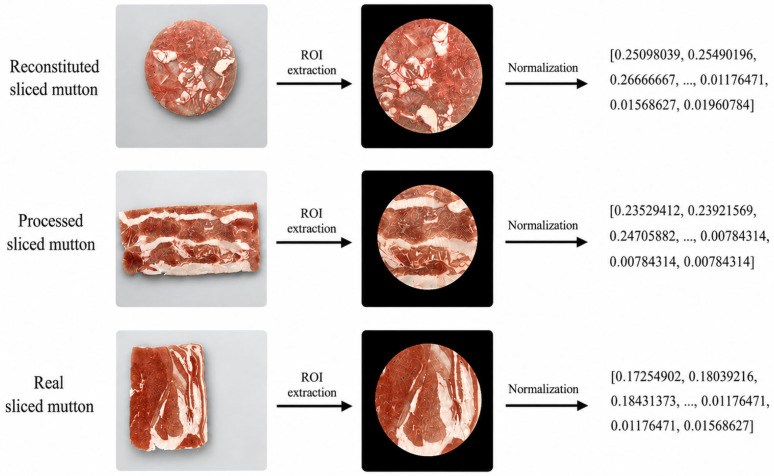
Image preprocessing workflow used for machine learning and deep learning analysis.

The image dataset was divided into training, validation, and testing subsets using a stratified sampling strategy at ratios of 70%, 15%, and 15%, respectively. Data augmentation was applied only to the training subset. Random transformations, including rotation, scaling, color perturbation, and noise injection, were used to generate three additional images from each training sample [20]. As a result, the number of training images increased from 420 to 1680, whereas the validation and testing subsets remained unchanged. All images were subsequently resized to the input dimensions required by the convolutional neural networks and normalized before model training. The overall preprocessing workflow is presented in Figure 2.

### 2.4. Color Feature Extraction

Color features are fundamental descriptors that characterize the distribution of color information in an image. The accurate extraction and analysis of color features are essential techniques in computer vision and image processing, forming the basis for various visual recognition and classification tasks [21,22,23,24,25,26]. Color models characterize the color information of samples from different perspectives. This study used color moments to quantify and extract features from three color spaces: RGB (Red Green Blue Color Model), HSV (Hue Saturation Value Color Model), and Lab (CIELAB Color Space). This extraction facilitated subsequent feature analysis and classification modeling. Specifically, the first-order moment (mean) represents the average brightness of the color channels, reflecting the concentration of the overall color. The second-order moment (standard deviation) measures the dispersion of color values. Higher values indicate more significant color variations and a broader distribution range. Color moments were calculated using the following methods [27]:

(1) $$\begin{array}{c} E_{i}=\frac{1}{N}\sum_{j=1}^{N}p_{ij} \end{array}$$

(2) $$\begin{array}{c} s_{i}={\left(\frac{1}{N-1}\sum_{j=1}^{N}{\left(p_{ij}-E_{i}\right)}^{2}\right)}^{\frac{1}{2}} \end{array}$$

where $E_{i}$ represents the mean pixel value of the i-th color channel, $s_{i}$ denotes the standard deviation of the color channel, $p_{ij}$ is the value of the j-th pixel in the i-th color channel, and N is the total number of pixels in each color channel.

### 2.5. Texture Feature Extraction

Texture features are a vital and straightforward group of image descriptors. In contrast to color features, texture offers a more detailed representation of the local spatial patterns in a picture. This study employed the Gray-Level Co-occurrence Matrix (GLCM) to examine the textural characteristics of mutton slices. The precise computation methodology is as follows:

For an input grayscale image f with horizontal and vertical pixel dimensions of $N_{y}$ and $N_{x}$, and the number of gray levels as $N_{g}$, the grayscale image f can be represented as:

(3) $$\begin{array}{c} f=\left\{G\left(x,y\right)|x=1,2,\ldots ,N_{x};y=1,2,\ldots ,N_{y};G=1,2,\ldots ,N_{g}\right\} \end{array}$$

Based on this, two external parameters are introduced: step length d and direction angle θ. Here, d represents the distance between point (*x*, *y*) and point $\left(x+dx,y+dy\right)$ in the coordinate system, and θ represents the angle between the line connecting these two points and the positive *x*-axis. Typically, the line along the *x*-axis is set to $\theta =0^{\circ }$, with counterclockwise being the positive direction. The GLCM $W\left(d,\theta \right)$ is defined as the probability of gray levels i and j appearing together at step length d and direction θ:

(4) $$\begin{array}{c} W\left(d,\;\theta \right)=P\left(i,\;j,\;d,\;\theta \right) \end{array}$$

Since W(d,θ) is a symmetric matrix, it satisfies the following symmetry relationships: $W\left(d,0^{\circ }\right)=W^{T}\left(d,180^{\circ }\right)$, $W\left(d,45^{\circ }\right)=W^{T}\left(d,225^{\circ }\right)$, $W\left(d,90^{\circ }\right)=W^{T}\left(d,270^{\circ }\right)$, $W\left(d,135^{\circ }\right)=W^{T}\left(d,315^{\circ }\right)$. Therefore, θ usually only at 0°, 45°, 90°, and 135° are considered. The probability (I,j,d,θ) of pixel pairs in GLCM is calculated as follows:

(5) $$\begin{array}{c} P\left(i,j,d,0^{\circ }\right)=\sum_{\left[\left(k,l\right),\left(m,n\right)\right]\in \left(L_{x}\times L_{y}\right)}\left[m-k=d,n-l=0,f\left(k,l\right)=i,f\left(m,n\right)=j\right] \end{array}$$

(6) $$\begin{array}{c} P\left(i,j,d,45^{\circ }\right)=\sum_{\left[\left(k,l\right),\left(m,n\right)\right]\in \left(L_{x}\times L_{y}\right)}\left[m-k=d,n-l=-d,f\left(k,l\right)=i,f\left(m,n\right)=j\right] \end{array}$$

(7) $$\begin{array}{c} P\left(i,j,d,90^{\circ }\right)=\sum_{\left[\left(k,l\right),\left(m,n\right)\right]\in \left(L_{x}\times L_{y}\right)}\left[m-k=0,n-l=d,f\left(k,l\right)=i,f\left(m,n\right)=j\right] \end{array}$$

(8) $$\begin{array}{c} P\left(i,j,d,135^{\circ }\right)=\sum_{\left[\left(k,l\right),\left(m,n\right)\right]\in \left(L_{x}\times L_{y}\right)}\left[m-k=-d,n-l=d,f\left(k,l\right)=i,f\left(m,n\right)=j\right] \end{array}$$

where (*k*, *l*) are the coordinates of the reference pixel in the co-occurrence matrix; (*m*, *n*) correspond to the neighboring pixel with direction d and distance θ from the reference pixel; $L_{x}\times L_{y}$ denotes the spatial extent of the image (width × height), i.e., the set of all pixel coordinates.

Different feature parameters convey distinct texture information. In this study, five parameters—Contrast, Correlation, Homogeneity, Energy, and Entropy—were selected for analysis. Their specific meanings and formulas are detailed in Ref. [28].

Contrast measures the extent of gray-level variations in the image. Higher contrast indicates more intense gray-level changes and rougher textures.

$$Contrast=\sum_{i=0}^{N_{g}-1}\sum_{j=0}^{N_{g}-1}{\left(i-j\right)}^{2}P\left(i,j\right)$$

Correlation measures the degree of linear correlation of gray levels in the image, i.e., the correlation of gray values between pixel pairs. Higher correlation signifies more regular image structures.

$$Correlation=\sum_{i=0}^{N_{g}-1}\sum_{j=0}^{N_{g}-1}\frac{\left(i-\mu_{i}\right)\left(j-\mu_{j}\right)P\left(i,j\right)}{\sigma_{i}\sigma_{j}}$$

where $\mu_{I}$ and $\mu_{j}$ are the means of the row and column gray levels, respectively; $\sigma_{i}$ and $\sigma_{j}$ are the standard deviations of the row and column gray levels.

Homogeneity measures the concentration of elements near the diagonal in the image’s gray-level matrix. A higher value indicates smaller gray-level differences in the neighborhood and smoother textures.

$$Homogeneity=\sum_{i=0}^{N_{g}-1}\sum_{j=0}^{N_{g}-1}\frac{P\left(i,j\right)}{1+{\left(i-j\right)}^{2}}$$

Energy is used to measure the uniformity of image texture. Higher energy indicates more consistent textures.

$$Energy=\sum_{i=0}^{N_{g}-1}\sum_{j=0}^{N_{g}-1}P{\left(i,j\right)}^{2}$$

Entropy is used to measure the complexity of the gray-level distribution in the image. A higher entropy value indicates more complex textures and richer image information.

$$Entropy=-\sum_{i=0}^{N_{g}-1}\sum_{j=0}^{N_{g}-1}P\left(i,j\right)\backslash log\;P\left(i,j\right)$$

### 2.6. Statistical Analysis

The discriminative capability of handcrafted image features among different sliced mutton categories was investigated using statistical analysis. Only the training set was used throughout the analysis to prevent information leakage. Several extracted color and texture descriptors did not satisfy normality assumptions. Therefore, nonparametric methods were adopted. The Kruskal–Wallis test was applied to examine differences among real, processed, and reconstituted sliced mutton samples for each feature. Significant overall differences were identified at *p* < 0.05. Dunn’s post hoc test with Bonferroni correction was subsequently performed to determine significant pairwise differences between categories [29].

To quantify the magnitude of observed differences, effect size analysis was additionally performed. For the Kruskal–Wallis test, epsilon squared (ε^2^) was calculated as a measure of overall effect size:

$$\varepsilon^{2}=\frac{H-k+1}{N-k}$$

where H is the Kruskal–Wallis statistic, k is the number of groups, and N is the total number of observations.

For pairwise Dunn comparisons, effect size (r) was calculated as:

$$r=\frac{Z}{\sqrt{N}}$$

where Z is the standardized test statistic and N is the sample size.

Furthermore, principal component analysis (PCA) was performed following feature standardization to visualize sample distribution patterns and investigate the overall separability among sliced mutton categories. Statistical analyses were implemented using Python, and a significance level of *p* < 0.05 was adopted throughout the study.

### 2.7. Model Validation Strategy

To ensure robust model development and minimize the influence of sampling variability, machine learning models were evaluated using a repeated stratified 5-fold cross-validation framework repeated 20 times (5 × 20 CV). Stratified sampling preserved the proportional distribution of real, processed, and reconstituted sliced mutton samples within each fold.

During each iteration, four folds were used for model training and one fold was used for validation. The procedure was repeated until each fold had served as the validation subset once, and the entire process was subsequently repeated 20 times using different random partitions. Model performance was calculated as the average of all cross-validation iterations.

After cross-validation, the models were retrained using the complete training dataset and evaluated on the independent testing subset. The testing subset was excluded from feature analysis, model development, and validation procedures to provide an unbiased assessment of model generalization ability.

### 2.8. Feature Selection and Dimensionality Reduction

Feature selection and dimensionality reduction were performed before model development to improve feature interpretability and reduce redundant information. All feature processing procedures were conducted independently within each cross-validation iteration. Only the fold-specific training subset was used during model fitting to prevent information leakage. The Kruskal–Wallis statistic was used for feature selection. Handcrafted features were ranked according to their discriminatory capability among sliced mutton categories. Features with stronger discriminatory ability were prioritized. Candidate subsets containing different numbers of top-ranked features were subsequently evaluated during model development.

Selected features were standardized using z-score normalization implemented by the StandardScaler method. This procedure minimized scale-related variability among extracted features. Standardization parameters were estimated exclusively from the training subset and then applied to the corresponding validation subset.

Principal component analysis (PCA) was subsequently applied to the standardized features. PCA reduced feature redundancy and improved feature representation. The PCA model was fitted exclusively on the fold-specific training subset, and the learned transformation was subsequently applied to the corresponding validation subset. This strategy ensured methodological rigor and prevented information leakage. The number of retained principal components was automatically determined to preserve 95% cumulative explained variance. Feature selection frequency across repeated cross-validation iterations was additionally recorded. This analysis was used to evaluate feature stability and reproducibility.

### 2.9. Machine Learning Model Construction

Conventional machine learning relies on distinct feature sets to achieve robust image classification. In this study, four representative algorithms—KNN, LDA, SVM, and RF—were leveraged to perform discriminative analysis on the image dataset.

#### 2.9.1. K-Nearest Neighbor (KNN)

The K-Nearest Neighbor (KNN) algorithm is a classification algorithm based on the spatial proximity of test and training samples. The algorithm determines the K nearest neighbors for each unclassified data item by computing their distances and then applies a category label using a majority voting process. KNN is classified as a typical lazy learning algorithm because it does not construct an explicit model during training, but instead relies on stored instances to perform prediction directly [30]. Although computationally straightforward and intuitive, it is mostly appropriate for small-scale datasets. KNN is widely used in practical applications for low-dimensional classification tasks, frequently with dimensionality reduction to enhance computational performance.

#### 2.9.2. Linear Discriminant Analysis (LDA)

Linear Discriminant Analysis (LDA) is a common supervised learning method designed to identify the optimal projection direction using the Fisher discriminant criterion. This approach minimizes within-class variance to cluster samples of the same category while maximizing between-class variance to separate different classes [31]. By incorporating class label information during dimensionality reduction, LDA maintains high discriminative capacity in low-dimensional spaces. For complex or non-linearly separable datasets, LDA is often combined with kernel methods (Kernel LDA) or integrated with deep learning models to enhance its modeling of intricate data structures.

#### 2.9.3. Support Vector Machine (SVM)

Support Vector Machine (SVM) constructs an optimal hyperplane to separate data points of different categories as much as possible in the feature space [32]. SVM maximizes the distance between data points and the hyperplane by identifying the hyperplane with the greatest margin, hence improving the generalization ability of the classification. SVM is applicable to both linear and nonlinear classification tasks. When data is not linearly separable, SVM uses kernel functions to transform the data into a high-dimensional feature space, rendering it linearly separable in that context. Currently, similar samples are in nearness, but examples from distinct categories are more distant, enabling the identification of the ideal classification hyperplane [33].

#### 2.9.4. Random Forest (RF)

Random Forest (RF) is an ensemble learning algorithm consisting of multiple independent decision trees [34]. RF utilizes Bagging to process data by randomly sampling subsets from the original dataset, ensuring diversity among the training data for each decision tree. During tree construction, RF introduces randomness in feature selection by selecting a subset of features to split decision nodes, thereby reducing correlation between trees. Final predictions are determined via majority voting for classification or averaging for regression [26]. The high generalization capacity of RF ensures robust performance when processing high-dimensional data and complex tasks.

### 2.10. Construction of Deep Learning Models

The capability of automatic feature learning for sliced mutton classification was further investigated using deep learning models. These models were developed directly from image data rather than handcrafted features. Unlike conventional machine learning approaches that depend on manually extracted color and texture descriptors, convolutional neural networks (CNNs) automatically learn hierarchical visual representations from raw image inputs. Transfer learning was adopted because of the relatively limited dataset size available in this study. This strategy improved feature extraction efficiency and accelerated model convergence.

**Figure 3:**
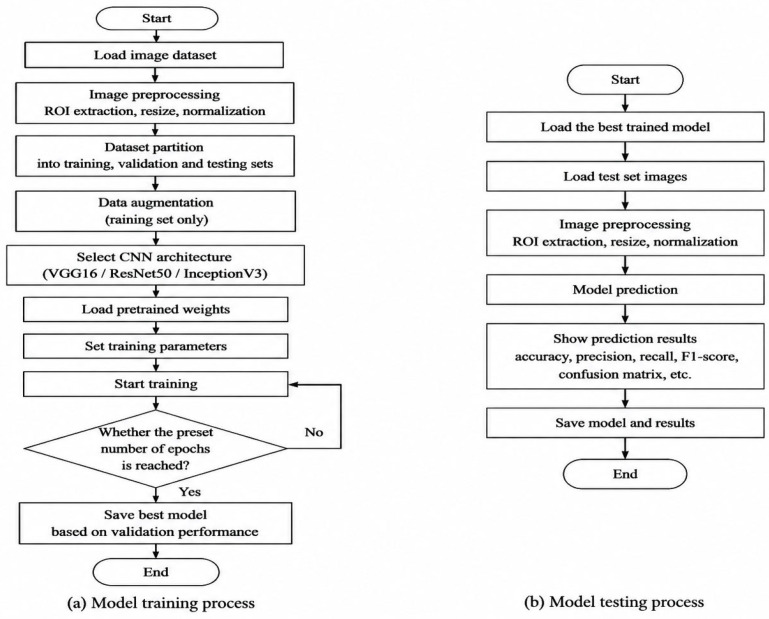
Workflow of the deep learning-based classification framework for sliced mutton recognition. (**a**) Model training workflow, including image preprocessing, dataset partitioning, data augmentation, transfer learning, network training, and optimal model selection. (**b**) Model testing workflow, including model deployment, image prediction, performance evaluation, and result generation.

The overall deep learning workflow is illustrated in Figure 3. Following image preprocessing, the dataset was divided into training, validation, and testing subsets using a stratified sampling strategy. Data augmentation was applied only to the training subset. This procedure increased image diversity and reduced overfitting. The augmented images were subsequently used for transfer learning-based CNN training.

During model development, pretrained convolutional backbones were initialized with ImageNet weights and fine-tuned for the three-class classification task. Model performance was monitored using the validation subset. The optimal model was selected according to validation performance and subsequently evaluated using the independent testing subset.

Three representative CNN architectures, VGG16, ResNet50, and InceptionV3, were evaluated in this study. VGG16 consists of sequential convolutional blocks and represents a classical deep convolutional architecture. ResNet50 incorporates residual connections, facilitating gradient propagation and deep feature learning. InceptionV3 employs multi-scale convolutional modules to improve feature extraction efficiency while reducing computational cost. These architectures differ substantially in network design and complexity. Therefore, they were selected to comprehensively assess the effectiveness of transfer learning for sliced mutton classification [35].

For model training, ImageNet-pretrained convolutional backbones were used for feature extraction. Task-specific fully connected layers were appended for three-class classification. Model optimization was performed using the Adam optimizer. Early stopping, adaptive learning-rate reduction, and model checkpointing were implemented to improve convergence stability and reduce overfitting. The validation subset was used for model selection and hyperparameter tuning. The independent testing subset was reserved exclusively for final model evaluation. All deep learning models were implemented using TensorFlow and trained on a workstation equipped with an Intel Core i7 processor and an NVIDIA GeForce RTX 4060 Laptop GPU with 13.7 GB of available graphics memory. Model training and inference were accelerated using GPU-based computation through the DirectML backend. The computational complexity and deployment efficiency of the evaluated CNN architectures were further assessed by comparing the number of model parameters, storage size, training time, and inference speed.

Gradient-weighted Class Activation Mapping (Grad-CAM) was subsequently employed to improve model interpretability. Grad-CAM visualizes image regions that contribute most strongly to model predictions. This analysis helped determine whether the networks focused on relevant morphological and textural characteristics of sliced mutton during classification. It also facilitated biological interpretation of the learned visual representations.

### 2.11. Calculations and Performance Evaluation

Accuracy, precision, recall, and F1-score were selected to evaluate the machine learning model performance in the adulterated mutton classification task.

Accuracy: The proportion of correctly predicted samples out of the total samples.

$$Accuracy=\frac{T_{p}}{N}$$

Precision: The proportion of true positive samples among those predicted as positive by the model.

$$Precision=\frac{T_{p}}{T_{p}+F_{p}}$$

Recall: The proportion of actual positive samples that are correctly predicted as positive.

$$Recall=\frac{T_{p}}{T_{p}+F_{n}}$$

F1-score: The harmonic mean of Precision and Recall.

$$F1=\frac{2\times Precision\times Recall}{Precision+Recall}$$

$T_{p}$ denotes the number of true positive samples, representing positive instances correctly identified by the model; $F_{p}$ indicates the number of false positive samples, where negative instances are misclassified as positive; $F_{n}$ represents the number of false negative samples, referring to positive instances incorrectly identified as negative; N denotes the total sample size.

## 3. Results and Analysis

### 3.1. Extraction of Color Features

Color features serve as critical descriptors for distinguishing among the three types of mutton slices. In this study, the mean and standard deviation of each channel within the RGB, HSV, and Lab color spaces were extracted as the primary color features. The results are summarized in Table 2.

According to Table 2, real sliced mutton exhibited the highest mean values across color channels, indicating a redder and brighter appearance. This reflects the natural and fresh characteristics of pure meat products. Real sliced mutton also showed the highest standard deviations, likely due to the significant color contrast between muscle and adipose tissues. In contrast, reconstituted sliced mutton had the lowest R, G, and B means, suggesting a dimmer appearance with insufficient redness. Its S, V, and b means were also the lowest, reflecting weaker color saturation and brightness. The minimal standard deviations in most channels for reconstituted sliced mutton indicated a more uniform color distribution, potentially resulting from the mixing and pressing of fat and muscle during production. Processed sliced mutton displayed color features intermediate between real and reconstituted types, showing closer proximity to the latter due to similar processing workflows.

### 3.2. Extraction of Texture Features

In this study, homogeneity, contrast, correlation, energy, and entropy were extracted as texture features for the mutton slices (Table 3). Real sliced mutton exhibited the highest energy and entropy, indicating rich texture information and complex internal structures. This corresponds to the clear and distinct grain characteristic of intact muscle tissue. Conversely, reconstituted sliced mutton showed the highest homogeneity and correlation, suggesting a smooth texture with high directional consistency. Its low entropy and energy reflected minimal texture variation and a lack of natural muscle grain. Most texture indicators for processed sliced mutton were intermediate between the real and reconstituted types. This suggests that while partial natural texture is retained, it is modified by mixing and seasoning during processing.

### 3.3. Statistical Feature Evaluation and Principal Component Analysis

**Figure 4:**
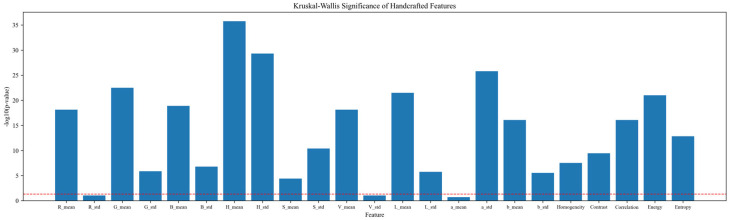
Statistical significance of handcrafted image features in the training subset based on the Kruskal–Wallis test.

Statistical analysis of the training subset showed significant differences in most handcrafted color and texture descriptors among the three sliced mutton categories (Figure 4). Features derived from the RGB, HSV, and Lab color spaces, together with gray-level co-occurrence matrix (GLCM) texture descriptors, exhibited strong discriminatory capability. Among them, H_mean, H_std, a_std, Energy, and Correlation produced the highest Kruskal–Wallis significance levels. These features effectively distinguished differences in color characteristics and surface texture patterns among categories. In contrast, descriptors such as a_mean and V_std showed relatively limited discriminatory power.

**Figure 5:**
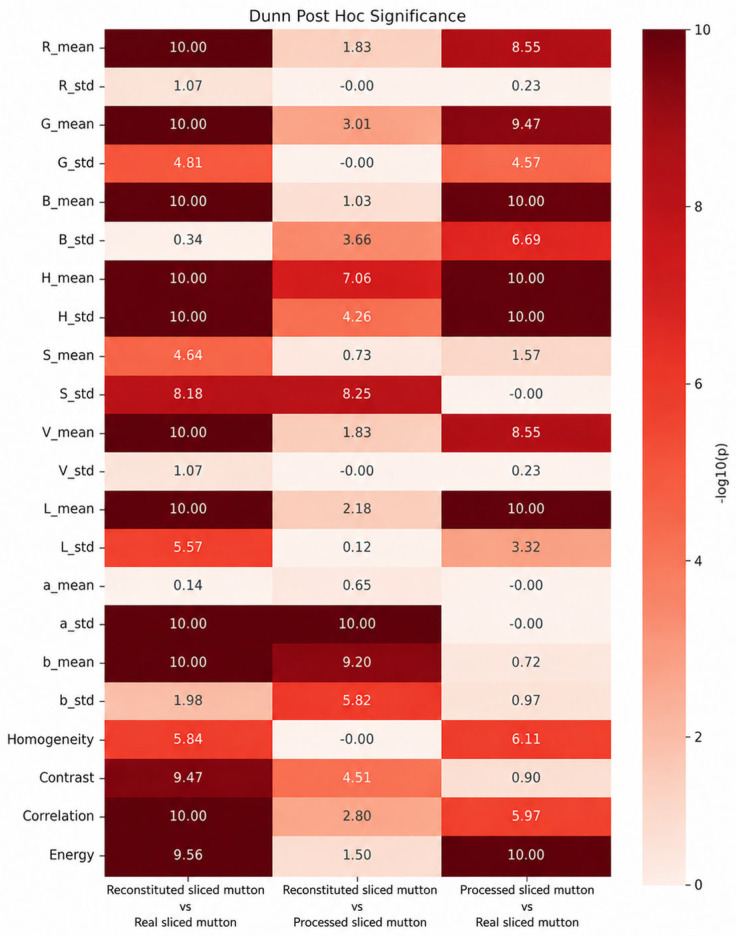
Dunn’s post hoc pairwise significance heatmap of handcrafted features.

Pairwise class separability was further evaluated using Dunn’s post hoc test with Bonferroni correction (Figure 5). The largest number of significant differences was observed between reconstituted sliced mutton and the other two categories. Multiple color and texture descriptors reached extremely high significance levels. These results indicate that reconstituted sliced mutton possessed distinct visual characteristics. In contrast, processed sliced mutton and real sliced mutton exhibited weaker separability for several descriptors, particularly those derived from the Lab color space and selected texture features. The greater similarity between these two categories may partially explain the higher misclassification rates observed in subsequent classification analyses.

**Figure 6:**
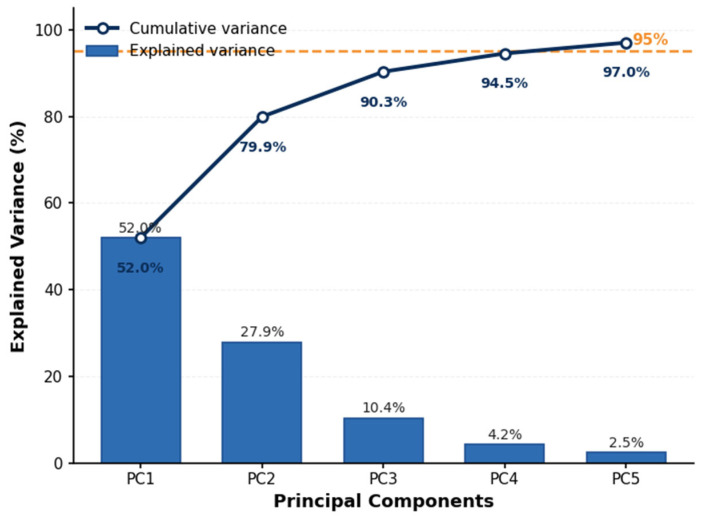
PCA scree plot derived from the training subset.

Principal component analysis (PCA) was applied after feature standardization to reduce feature redundancy and multicollinearity. PCA fitting was performed exclusively within each training fold during cross-validation to prevent information leakage. As shown in Figure 6, the first two principal components explained 79.9% of the total variance, whereas five principal components retained 97.0% cumulative variance. These findings suggest substantial correlation among the original 23 handcrafted descriptors. Most of the feature information could therefore be represented within a low-dimensional feature space.

### 3.4. Machine Learning Classification Performance

#### 3.4.1. Model Comparison and Selection Based on Repeated Cross-Validation

The classification performance of handcrafted image features was evaluated using four conventional machine learning algorithms, including Random Forest (RF), Support Vector Machine (SVM), K-Nearest Neighbor (KNN), and Linear Discriminant Analysis (LDA). Model development and evaluation were performed within a repeated stratified 5 × 20 cross-validation (CV) framework. Feature selection, standardization, and principal component analysis (PCA) were conducted independently within each training fold to prevent information leakage.

The cross-validation and validation results are summarized in Table 4. Among the evaluated models, SVM achieved the highest classification performance, with a repeated CV accuracy of 87.48 ± 3.05% and a validation accuracy of 85.56%. RF showed comparable performance, reaching a repeated CV accuracy of 86.79 ± 3.12% and a validation accuracy of 84.44%. Lower accuracies were obtained for KNN and LDA, which achieved repeated CV accuracies of 84.44 ± 3.30% and 82.14 ± 2.98%, respectively. The relatively small standard deviations across repeated runs indicate good model stability and reproducibility.

The superior performance of SVM suggests that the relationships among handcrafted image features were not fully described by linear decision boundaries. This observation is further supported by the lower performance of LDA, which assumes linear class separation. Similar trends have been reported in previous image-based food classification studies, where nonlinear classifiers generally exhibited stronger discrimination capability than linear models.

Although the optimal number of selected features varied slightly across cross-validation folds, SVM consistently maintained strong classification performance. RF also achieved competitive results throughout model development. Therefore, both SVM and RF were retained for subsequent independent test evaluation to further assess model generalization on unseen samples.

#### 3.4.2. Independent Test Evaluation

Following model selection, the two best-performing classifiers, Random Forest (RF) and Support Vector Machine (SVM), were retrained using the combined training and validation subsets (n = 510). Model performance was subsequently evaluated using an independent testing subset (n = 90). Testing samples were excluded from feature selection, parameter optimization, and model development to ensure an unbiased evaluation of model generalization.

**Figure 7:**
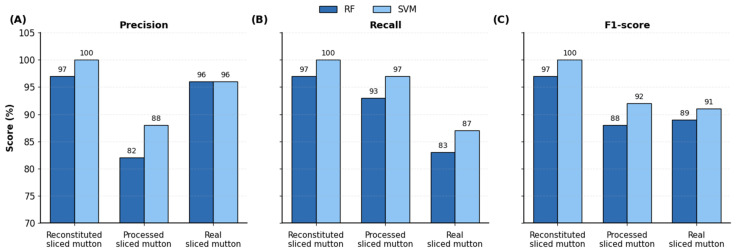
Class-wise performance comparison of the Random Forest (RF) and Support Vector Machine (SVM) classifiers on the independent test set. (**A**) Precision, (**B**) Recall, and (**C**) F1-score.

The predictive performances of RF and SVM on the independent testing subset are summarized in Figure 7. Both classifiers achieved high classification accuracy, demonstrating the effectiveness of handcrafted color and texture descriptors for sliced mutton authentication. RF achieved an overall accuracy of 91.11%, with weighted precision, recall, and F1-score values of 91.72%, 91.11%, and 91.15%, respectively. SVM achieved a higher overall accuracy of 94.44%, together with weighted precision, recall, and F1-score values of 94.73%, 94.44%, and 94.43%, respectively. Bootstrap resampling (1000 iterations) further confirmed stable predictive performance, yielding estimated 95% confidence intervals of 84.44–96.67% for RF and 88.89–98.89% for SVM.

Class-wise performance metrics are presented in Figure 7. Both classifiers achieved excellent recognition performance for reconstituted sliced mutton. Notably, SVM reached 100% precision, recall, and F1-score for this category. The largest performance difference between the two models was observed for processed sliced mutton. Compared with RF, SVM increased precision from 82% to 88%, recall from 93% to 97%, and F1-score from 88% to 92%. These results indicate improved discrimination of this visually similar category. For real sliced mutton, both classifiers achieved high precision, whereas SVM maintained slightly higher recall and F1-score values.

**Figure 8:**
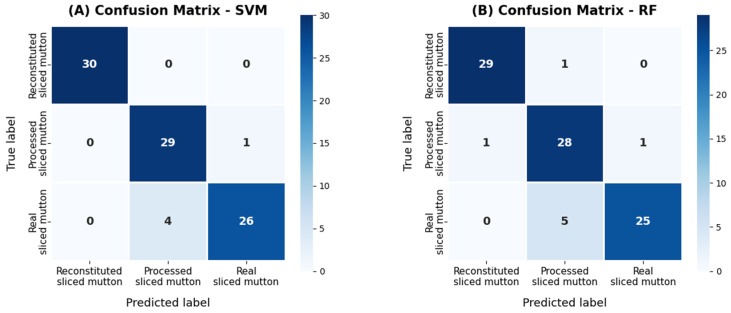
Confusion matrices of the two best-performing machine learning classifiers on the independent test set. (**A**) Support Vector Machine (SVM) and (**B**) Random Forest (RF). The independent test set consisted of 90 samples, including 30 reconstituted sliced mutton, 30 processed sliced mutton, and 30 real sliced mutton samples.

The confusion matrices obtained from the independent testing subset are presented in Figure 8. Both models correctly classified all or nearly all reconstituted sliced mutton samples, highlighting the distinct visual characteristics of this category. In contrast, most classification errors occurred between processed sliced mutton and real sliced mutton. For SVM, only five samples were misclassified between these two categories, and no confusion was observed between reconstituted and real sliced mutton. RF exhibited additional misclassifications involving reconstituted sliced mutton, indicating less distinct class separation and lower overall classification performance.

The misclassification patterns were consistent with the statistical feature analysis presented in Section 3.4. Dunn’s post hoc test revealed significant differences between reconstituted sliced mutton and the other two categories across multiple color and texture descriptors. In contrast, processed sliced mutton and real sliced mutton exhibited weaker feature separability. Similar trends were observed in the confusion matrices, where most prediction errors were concentrated between these two categories. These findings suggest that the visual characteristics of processed sliced mutton and real sliced mutton overlapped to a greater extent than those of reconstituted sliced mutton, contributing to the increased classification difficulty.

Overall, both RF and SVM demonstrated strong predictive capability for sliced mutton authentication using handcrafted image descriptors. However, SVM achieved consistently superior performance on the independent testing subset and exhibited fewer class-level misclassifications. Therefore, SVM was selected as the optimal machine learning model for subsequent misclassification analysis under the present experimental conditions.

#### 3.4.3. Misclassification Analysis

The independent testing results showed that the optimal SVM classifier misclassified only five of the 90 testing samples, corresponding to a misclassification rate of 5.56%. All classification errors occurred between processed sliced mutton and real sliced mutton, whereas reconstituted sliced mutton was completely separated from the other categories. This observation agrees with the statistical feature analysis in Section 3.4, which revealed greater feature similarity between processed and real sliced mutton than between either category and reconstituted sliced mutton.

**Figure 9:**
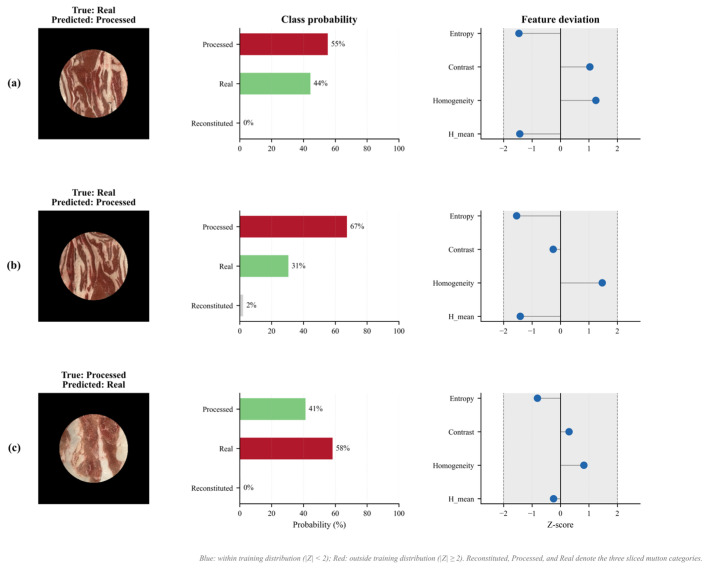
(**a**–**c**) Representative misclassified samples and feature deviation analysis of the optimal SVM classifier.

Representative misclassified samples were further analyzed using ROI appearance, class probability distributions, and standardized feature deviations (Figure 9). For all misclassified samples, the predicted probabilities of the true and assigned classes were relatively close. This result suggests that these samples were located near the decision boundary. For example, sample (a), a real sliced mutton sample misclassified as processed sliced mutton, received probabilities of 44% and 55% for the two categories, respectively. Similar patterns were observed for the remaining samples, where prediction confidence remained moderate despite incorrect classification.

Feature deviation analysis showed that misclassified samples frequently exhibited texture characteristics that differed from those of correctly classified samples within the same category. Several samples displayed elevated contrast and homogeneity values, reflecting altered local intensity variation and spatial uniformity. These deviations shifted the feature representations toward neighboring class distributions and increased the likelihood of misclassification.

**Figure 10:**
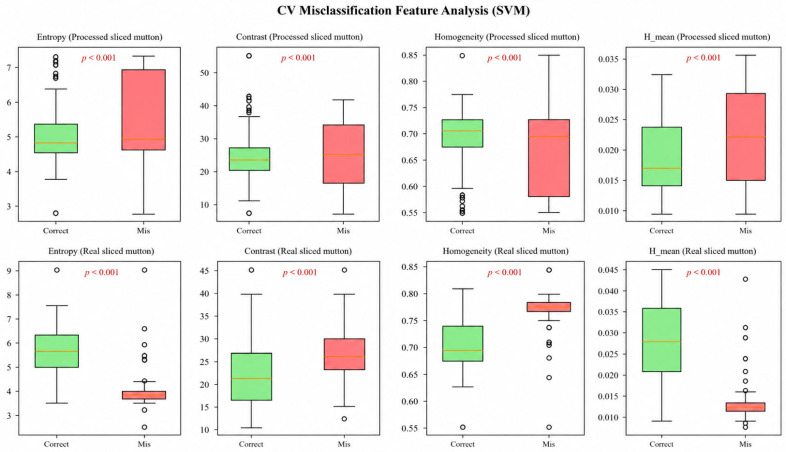
Texture feature deviations associated with SVM misclassification of processed and real sliced mutton samples.

To further evaluate these observations, the distributions of the four retained descriptors (Entropy, Contrast, Homogeneity, and H_mean) were compared between correctly classified and misclassified samples (Figure 10). Significant differences were observed for all four descriptors (*p* < 0.01). For processed sliced mutton, misclassified samples exhibited higher entropy, contrast, and H_mean values together with lower homogeneity, indicating texture characteristics more similar to those of real sliced mutton. Conversely, misclassified real sliced mutton samples showed lower entropy and H_mean values but higher homogeneity and contrast values than correctly classified samples. These findings indicate convergence of texture characteristics between the two categories and help explain the observed prediction errors.

Overall, the misclassification analysis suggests that classification errors were closely associated with shared texture characteristics between processed sliced mutton and real sliced mutton. Despite this partial similarity, the SVM classifier maintained high classification accuracy on the independent testing subset, supporting the effectiveness of the proposed machine vision approach for sliced mutton authentication.

### 3.5. Results of Deep Learning Models

#### 3.5.1. Model Training Dynamics and Convergence Analysis

**Figure 11:**
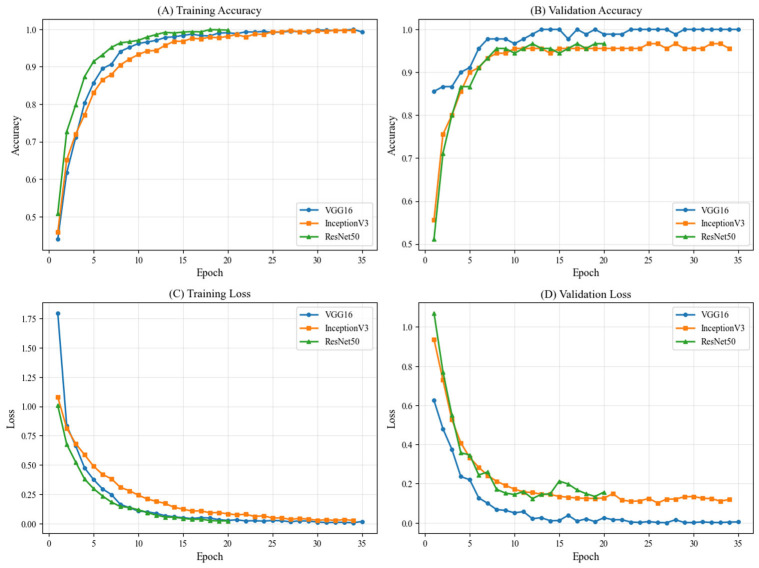
Training dynamics of the three transfer learning models during the classification of sliced mutton images.

The learning dynamics of VGG16, ResNet50, and InceptionV3 during transfer learning are presented in Figure 11. Training and validation accuracy curves, together with the corresponding loss curves, were used to evaluate model convergence behavior, optimization efficiency, and generalization capability.

As shown in Figure 11A,C, all three CNN architectures exhibited rapid improvements in training accuracy accompanied by substantial reductions in training loss during the early training stage, indicating effective adaptation of the ImageNet-pretrained weights to the sliced mutton classification task. The majority of performance gains were achieved within the first several epochs, after which the training curves gradually approached stable convergence. Among the evaluated models, VGG16 and ResNet50 reached high training accuracy at an earlier stage, whereas InceptionV3 required a longer optimization process before achieving comparable training performance.

The validation curves further highlighted differences in generalization capability among the three architectures (Figure 11B,D). VGG16 consistently achieved the highest validation accuracy while maintaining the lowest validation loss throughout most of the training process. After the initial learning stage, the validation accuracy rapidly approached saturation and remained highly stable, suggesting robust feature extraction and strong generalization ability. ResNet50 exhibited moderate fluctuations in both validation accuracy and validation loss despite achieving satisfactory overall performance. InceptionV3 showed slightly larger fluctuations in validation accuracy and maintained relatively higher validation loss values compared with VGG16; however, the model still converged successfully and achieved stable validation performance. Importantly, no obvious divergence between training and validation curves was observed for any of the three architectures. Validation accuracy remained stable while validation loss did not exhibit substantial increases during later training stages, indicating that the adopted transfer learning strategy, data augmentation procedures, and regularization mechanisms helped reduce the risk of severe overfitting.

To quantitatively evaluate the effect of data augmentation, an additional ablation experiment was performed using the VGG16 model. As shown in Table 5, data augmentation increased the number of training images from 420 to 1680 and improved classification accuracy from 94.34 ± 1.34% to 96.42 ± 0.51%. Similar improvements were observed for precision, recall, and F1-score, while the reduced standard deviations indicated enhanced model stability. These results confirm that data augmentation effectively improved the generalization capability of the CNN models under limited training data conditions.

#### 3.5.2. Classification Performance of Deep Learning Models

Following the convergence analysis, the classification performances of the three transfer learning models were further evaluated using an independent testing subset. The results are summarized in Table 5. Overall, all CNN architectures achieved satisfactory recognition performance for sliced mutton classification, demonstrating the effectiveness of deep learning-based feature extraction from smartphone-acquired images.

As shown in Table 6, VGG16 achieved the highest overall classification performance, yielding an accuracy of 96.42 ± 0.51%, precision of 96.56 ± 0.43%, recall of 96.42 ± 0.51%, and F1-score of 96.42 ± 0.50%. Furthermore, VGG16 exhibited the smallest standard deviations across repeated training runs, indicating superior model stability and reproducibility. ResNet50 achieved intermediate performance, whereas InceptionV3 produced the lowest classification accuracy and showed greater variability among repeated experiments.

The superior performance of VGG16 was consistent with the convergence behaviors observed in Figure 11. Although ResNet50 and InceptionV3 possess deeper and more sophisticated architectures, their increased model complexity did not translate into improved classification performance under the current dataset conditions. By contrast, the relatively simple sequential structure of VGG16 appeared more suitable for learning discriminative visual features from sliced mutton images, resulting in improved classification accuracy and enhanced generalization capability.

**Figure 12:**
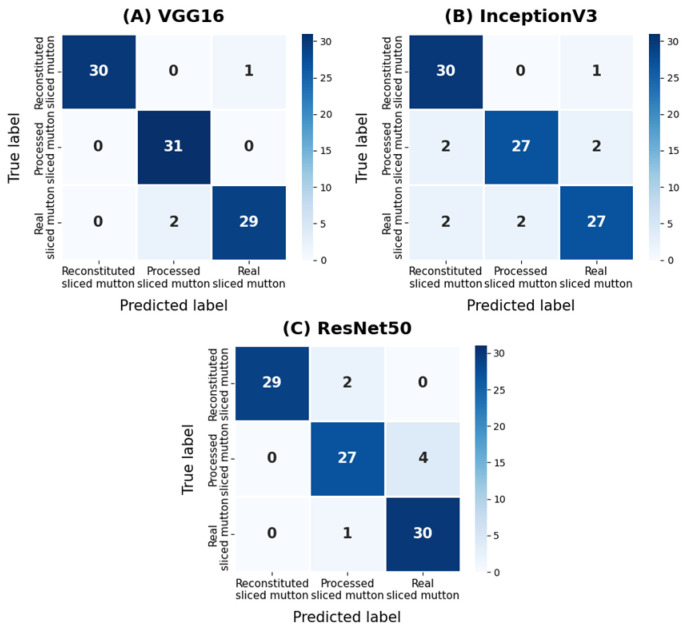
Confusion matrices of the three deep learning models for mutton slice classification. (**A**) VGG16, (**B**) InceptionV3, and (**C**) ResNet50. The independent test set consisted of 90 samples, including 30 reconstituted sliced mutton, 30 processed sliced mutton, and 30 real sliced mutton samples.

To further investigate classification behavior at the category level, confusion matrices were analyzed (Figure 12). As shown in Figure 12A, VGG16 demonstrated the highest discriminative ability, with only three misclassified samples among the entire testing subset. Processed sliced mutton achieved perfect classification performance, whereas only minor confusion was observed between reconstituted and real sliced mutton samples. These results indicate that VGG16 successfully captured subtle visual differences associated with tissue structure and fat distribution.

ResNet50 also exhibited satisfactory category-level recognition performance (Figure 12C). However, misclassification mainly occurred between processed sliced mutton and real sliced mutton, with several processed samples incorrectly predicted as real sliced mutton. Similarly, InceptionV3 displayed greater inter-class confusion than the other two models (Figure 12B), particularly between processed and real sliced mutton samples. These findings suggest that processed sliced mutton represented the most challenging category for classification, likely because processing operations partially altered the natural morphological characteristics of mutton and generated visual features shared with both real and reconstituted products.

The computational complexity and deployment efficiency of the three CNN architectures are summarized in Table 7. VGG16 contained 14.88 million parameters and achieved the highest classification accuracy, although it required a longer training time than InceptionV3. InceptionV3 exhibited the smallest model size and shortest training time, indicating superior computational efficiency. ResNet50 possessed the largest number of parameters and model size among the evaluated architectures but did not provide a corresponding improvement in classification performance. Despite these differences, all three models achieved rapid inference speeds, requiring less than 0.011 s per image, demonstrating their potential applicability for real-time meat authentication systems.

Overall, VGG16 provided the most favorable balance between classification accuracy, robustness, and computational efficiency. Combined with its stable convergence behavior and minimal inter-class confusion, VGG16 was identified as the optimal deep learning architecture for sliced mutton authentication in this study.

#### 3.5.3. Grad-CAM Visualization and Feature Interpretation

**Figure 13:**
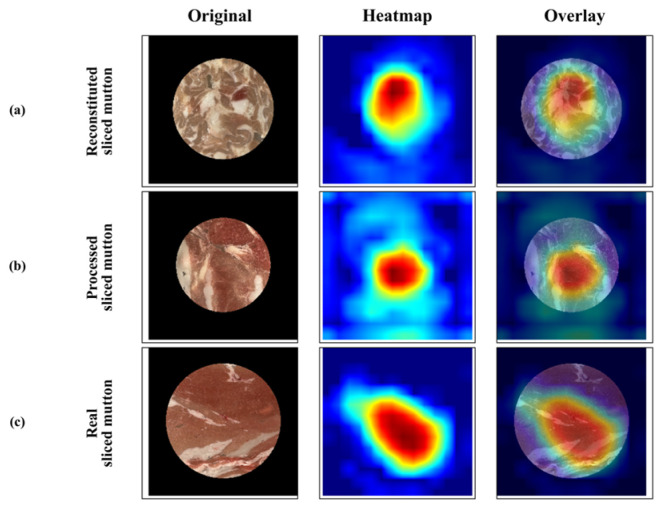
Representative Grad-CAM visualizations generated by the optimal VGG16 model for the classification of (**a**) reconstituted sliced mutton, (**b**) processed sliced mutton, and (**c**) real sliced mutton. The heatmaps highlight image regions contributing most strongly to model predictions, while the overlay images illustrate the correspondence between activation patterns and morphological structures of the samples.

To further investigate the visual features contributing to model predictions, Gradient-weighted Class Activation Mapping (Grad-CAM) was applied to the optimal VGG16 model. Representative activation maps for reconstituted, processed, and real sliced mutton samples are presented in Figure 13.

As shown in Figure 13, the activated regions were primarily concentrated within the sliced mutton tissues rather than the surrounding background. This observation indicates that the model mainly relied on information derived from the sample regions during classification. The activation patterns also suggest that the high classification performance of VGG16 was associated with intrinsic visual characteristics of sliced mutton rather than background information.

For reconstituted sliced mutton, strong activation was mainly observed in regions exhibiting heterogeneous fat distribution and irregular tissue organization (Figure 13a). In processed sliced mutton samples, activation was concentrated around reconstructed muscle–fat interfaces (Figure 13b). These regions may represent important visual cues associated with processing-induced structural characteristics. For real sliced mutton, activation was predominantly localized along natural muscle-fiber boundaries and marbling structures (Figure 13c), reflecting characteristic tissue morphology.

The Grad-CAM visualizations indicate that VGG16 captured texture and structural information relevant to the discrimination of different sliced mutton categories. The activated regions corresponded closely to morphologically meaningful tissue structures. These observations support the interpretability of the deep learning model and provide additional insight into the visual features contributing to classification. Together with the high classification accuracy and stable model performance observed previously, the Grad-CAM results further support the applicability of VGG16 for smartphone-based sliced mutton authentication.

## 4. Discussion

The present study demonstrated that smartphone-acquired RGB images combined with machine learning and deep learning techniques can successfully discriminate among real, processed, and reconstituted sliced mutton. Statistical analysis revealed significant differences in multiple color and texture descriptors across categories. Texture-derived features generally exhibited stronger discriminatory capability than color descriptors. This result indicates that differences in muscle-fiber organization, fat distribution, and tissue morphology played important roles in category discrimination. Similar observations have been reported in previous machine vision studies of meat products, where texture information often provides more robust classification cues than color characteristics alone.

Principal component analysis further supported the effectiveness of the extracted handcrafted features. Although partial overlap was observed among categories, particularly between processed and real sliced mutton, the overall distribution patterns remained distinguishable in the reduced feature space. This overlap was consistent with the statistical feature analysis and subsequent classification results. Processed sliced mutton represented the most challenging category because its visual characteristics shared similarities with both real and reconstituted sliced mutton. Unlike reconstituted products, which typically exhibit relatively homogeneous tissue organization following restructuring, processed sliced mutton often retains portions of natural muscle morphology while displaying processing-induced structural modifications. Consequently, processed sliced mutton occupied an intermediate position between the other two categories, resulting in greater classification ambiguity.

Among the evaluated machine learning algorithms, SVM achieved the highest classification performance. The model consistently outperformed RF, KNN, and LDA during repeated cross-validation and independent testing. Misclassification analysis showed that most prediction errors occurred between processed sliced mutton and real sliced mutton, whereas reconstituted sliced mutton was recognized with comparatively high accuracy. The confusion matrix analysis further confirmed this pattern. Samples that were incorrectly classified generally exhibited class probabilities close to the decision boundary, suggesting substantial similarity in their visual characteristics. Moreover, feature deviation analysis revealed that misclassified samples frequently displayed atypical texture characteristics compared with correctly classified samples from the same category. These observations further highlight the importance of texture information and help explain the reduced separability between processed and real sliced mutton.

Deep learning models achieved higher classification performance than conventional machine learning approaches. Unlike handcrafted-feature-based models, convolutional neural networks automatically learned hierarchical visual representations directly from image data. Among the evaluated architectures, VGG16 achieved the highest classification accuracy and exhibited the most stable convergence behavior. Although ResNet50 and InceptionV3 possess greater architectural complexity, their advantages were not fully realized under the current dataset conditions. The relatively simple architecture of VGG16 may have been better suited to the scale of the current dataset, resulting in improved classification performance. Furthermore, no obvious divergence between training and validation loss curves was observed, suggesting a relatively low risk of severe overfitting under the adopted training strategy.

Grad-CAM visualization provided additional insight into the decision-making process of the deep learning models. Activated regions were primarily concentrated within sliced mutton tissues rather than background areas. The highlighted regions corresponded closely to morphologically meaningful structures, including muscle–fat interfaces, marbling patterns, and tissue boundaries. Distinct activation patterns were observed among the three sliced mutton categories, reflecting differences in tissue organization and structural characteristics. These observations suggest that the CNN models primarily focused on morphologically meaningful tissue regions during classification. The Grad-CAM results therefore improve the interpretability of the developed deep learning framework and support the interpretability of the learned visual representations.

Despite the encouraging results, several limitations should be acknowledged. First, sample categories were assigned according to product labeling information, ingredient declarations, processing descriptions, and corresponding execution standards provided by manufacturers. No independent molecular, spectroscopic, or compositional verification was performed. Therefore, the established dataset reflects manufacturer-declared commercial classifications and should be regarded as suitable for preliminary screening studies rather than definitive authentication. Future studies should incorporate complementary analytical techniques, such as DNA-based methods, spectroscopic analysis, or compositional profiling, to establish more rigorously validated reference datasets.

Second, all images were acquired using a single smartphone model (iPhone 15) under standardized laboratory conditions. Although this configuration ensured experimental consistency, the robustness of the proposed framework across different smartphone cameras, image sensors, and device manufacturers remains to be evaluated. Additional validation using multiple imaging devices will be necessary before broader practical deployment can be considered.

Third, image acquisition was conducted under controlled illumination conditions using a dedicated imaging chamber. While such conditions are appropriate for methodological development, model performance under more complex real-world environments, including supermarkets, restaurants, retail markets, and household settings, remains unknown. Future studies should investigate illumination variability and environmental influences to improve the generalizability of the framework.

Fourth, the present dataset was collected from a limited number of commercial brands and product categories. Although repeated cross-validation and independent testing were performed, broader validation involving additional manufacturers, production batches, geographical regions, and adulteration scenarios will be necessary to further assess model robustness and generalization capability.

Overall, the proposed smartphone-based machine vision framework demonstrates considerable potential as a rapid, low-cost, and non-destructive screening tool for differentiating real, processed, and reconstituted sliced mutton. Additional validation using more diverse datasets, practical imaging conditions, and independent authentication methods will be necessary before routine implementation. Future studies integrating machine vision with spectroscopic, molecular, or chemical information may further improve classification robustness and support the development of more reliable meat authenticity assessment systems.

## 5. Conclusions

This study developed a low-cost, non-destructive, smartphone-based image recognition framework for the authentication of real, processed, and reconstituted sliced mutton. A total of 600 images were analyzed using a workflow integrating image preprocessing, handcrafted feature extraction, statistical analysis, machine learning, and deep learning. Statistical analysis demonstrated significant differences in multiple color and texture descriptors among categories, while principal component analysis confirmed the feasibility of distinguishing the three sliced mutton types using image-derived features. Among the evaluated machine learning models, Support Vector Machine (SVM) achieved the best classification performance. For deep learning, transfer learning-based VGG16, ResNet50, and InceptionV3 models were developed, with VGG16 achieving the highest classification accuracy (96.42 ± 0.51%) and the most stable training performance.

The results indicate that texture and structural characteristics play important roles in sliced mutton discrimination. Processed sliced mutton exhibited greater similarity to real sliced mutton than to reconstituted sliced mutton, resulting in increased classification difficulty. Grad-CAM visualizations further indicated that the deep learning models primarily focused on morphologically meaningful tissue regions associated with muscle and fat distribution, supporting the interpretability of the classification results.

Overall, the proposed framework demonstrates considerable potential for rapid and portable meat authentication without requiring specialized laboratory equipment or destructive sample preparation. The integration of smartphone imaging and artificial intelligence provides a practical solution for preliminary screening and on-site inspection within meat supply chains and retail markets. Future studies should expand sample diversity, evaluate model robustness under more diverse imaging conditions, and explore multimodal approaches integrating machine vision with spectroscopic or compositional information to further improve authentication reliability and practical applicability.

## Figures and Tables

**Table 1:** Classification basis for sliced mutton in this study.

Category	Ingredient Composition	Standard
Real sliced mutton	Mutton	GB2707-2016
Processed sliced mutton	Mutton + Water + Additives (edible salt, carrageenan, etc.)	SB/T10379-2012
Reconstituted sliced mutton	Duck meat + Sheep fat + Water + Additives	SB/T10482-2008

**Table 2:** The average color characteristics of three types of sliced mutton in three color spaces.

Features	Reconstituted Sliced Mutton	Processed Sliced Mutton	Real Sliced Mutton
R mean	45.63	46.14	57.66
G mean	23.72	25.75	28.2
B mean	55.34	57.06	59.39
H mean	2.87	2.77	3.13
S mean	45.63	46.15	57.67
V mean	56.37	57.38	72.13
L mean	36.91	37.6	44.74
a mean	134.5	133.88	136.41
b mean	9.03	9.28	14.45
R SD	31.37	32.06	35.48
G SD	78.6	77.41	84.83
B SD	43.39	47.32	50.28
H SD	32.07	32.39	44.15
S SD	5	5.53	5.61
V SD	78.6	77.41	84.83
L SD	132.76	132.9	135.96
a SD	64.06	64.61	68.23
b SD	11.33	10.15	13.41

**Table 3:** The average texture features of the three types of sliced mutton.

Features	Reconstituted Sliced Mutton	Processed Sliced Mutton	Real Sliced Mutton
Homogeneity	0.89	0.87	0.86
Contrast	1.18	1.90	1.78
Correlation	0.96	0.93	0.94
Energy	2.25	2.48	2.92
Entropy	1.52	1.68	1.96

**Table 4:** Classification performance of four machine learning models based on repeated cross-validation and validation evaluation. Error bars represent standard deviations from repeated 5-fold cross-validation (20 repeats).

Model	Repeated CV Accuracy (%)	Validation Accuracy (%)	Validation F1(%)
RF	86.79 ± 3.12	84.44	84.13
SVM	87.48 ± 3.05	85.56	85.58
KNN	84.44 ± 3.30	84.44	84.38
LDA	82.14 ± 2.98	83.78	83.62

**Table 5:** Effect of data augmentation on the classification performance of the VGG16 model for sliced mutton authentication.

Training Strategy	Training Images	Accuracy (%)	Precision (%)	Recall (%)	F1-Score (%)
With augmentation	1680	96.42 ± 0.51	96.56 ± 0.43	96.42 ± 0.51	96.42 ± 0.50
Without augmentation	420	94.34 ± 1.34	94.55 ± 1.08	94.34 ± 1.34	94.33 ± 1.35

**Table 6:** Performance comparison of the three transfer learning-based CNN architectures for sliced mutton classification.

Model	Accuracy (%)	Precision (%)	Recall (%)	F1-Score (%)
VGG16	96.42 ± 0.51	96.56 ± 0.43	96.42 ± 0.51	96.42 ± 0.50
InceptionV3	88.53 ± 1.64	88.62 ± 1.70	88.53 ± 1.64	88.47 ± 1.64
ResNet50	91.40 ± 1.08	91.53 ± 1.16	91.40 ± 1.08	91.37 ± 1.09

**Table 7:** Computational efficiency and model complexity of the three deep learning models.

Model	Parameters (M)	Model Size (MB)	Training Time (s)	Testing Time (s/Image)
VGG16	14.88	177.29	177.29	0.0071
InceptionV3	22.36	145.22	113.41	0.0102
ResNet50	24.15	207.20	117.13	0.0096

## Data Availability

The original contributions presented in the study are included in the article. Further inquiries can be directed to the corresponding author.
